# Food quality and quantity are more important in explaining foraging of an intermediate‐sized mammalian herbivore than predation risk or competition

**DOI:** 10.1002/ece3.4372

**Published:** 2018-07-24

**Authors:** Martijn J. A. Weterings, Sander Moonen, Herbert H. T. Prins, Sipke E. van Wieren, Frank van Langevelde

**Affiliations:** ^1^ Resource Ecology Group Wageningen University Wageningen The Netherlands; ^2^ Wildlife Management Department of Animal Management Van Hall Larenstein University of Applied Sciences Leeuwarden The Netherlands; ^3^ Institute of Avian Research Wilhelmshaven Germany; ^4^ School of Life Sciences University of KwaZulu‐Natal Durban South Africa

**Keywords:** accelerometer, GPS, herbivore, *Lepus europaeus*, plant resources, prey behavior, space use

## Abstract

During times of high activity by predators and competitors, herbivores may be forced to forage in patches of low‐quality food. However, the relative importance in determining where and what herbivores forage still remains unclear, especially for small‐ and intermediate‐sized herbivores. Our objective was to test the relative importance of predator and competitor activity, and forage quality and quantity on the proportion of time spent in a vegetation type and the proportion of time spent foraging by the intermediate‐sized herbivore European hare (*Lepus europaeus*). We studied red fox (*Vulpes vulpes*) as a predator species and European rabbit (*Oryctolagus cuniculus*) as a competitor. We investigated the time spent at a location and foraging time of hare using GPS with accelerometers. Forage quality and quantity were analyzed based on hand‐plucked samples of a selection of the locally most important plant species in the diet of hare. Predator activity and competitor activity were investigated using a network of camera traps. Hares spent a higher proportion of time in vegetation types that contained a higher percentage of fibers (i.e., NDF). Besides, hares spent a higher proportion of time in vegetation types that contained relatively low food quantity and quality of forage (i.e., high percentage of fibers) during days that foxes (*Vulpes vulpes*) were more active. Also during days that rabbits (*Oryctolagus cuniculus*) were more active, hares spent a higher proportion of time foraging in vegetation types that contained a relatively low quality of forage. Although predation risk affected space use and foraging behavior, and competition affected foraging behavior, our study shows that food quality and quantity more strongly affected space use and foraging behavior than predation risk or competition. It seems that we need to reconsider the relative importance of the landscape of food in a world of fear and competition.

## 1 INTRODUCTION

Decisions of animals about where and what to eat depend on the outcome of the costs and benefits of foraging (Robbins, 1993). Costs include searching and handling time of the food, the risk of predation (i.e., landscape of fear sensu Laundré, Hernández, & Altendorf, 2001), and the effects of competitors (Pays et al., 2012). Mammalian herbivores are predicted to select food patches that optimize intake rate (i.e., forage quantity) or digestible intake (i.e., forage quality) given these costs (Shipley, 2007). Especially for small‐ and intermediate‐sized mammalian herbivores, the trade‐off between predation risk and food intake is important as, on the one hand, these herbivores have low absolute nutritional requirements but need highly digestible food compared to large herbivores. As high‐quality food is often scarce, these herbivores have to spend a lot of time searching for patches with high‐quality food. On the other hand, small‐ and intermediate‐sized herbivores are more vulnerable for predation than larger ones, because they are often affected by multiple opportunistic predators (Sinclair, Mduma, & Brashares, 2003; Thaker et al., 2011). Populations of small‐ and intermediate‐sized herbivore species are suggested to be more strongly determined by predation than by food limitation (Brown & Kotler, 2004; Hopcraft, Olff, & Sinclair, 2010; Sinclair et al., 2003). In times of high predation risk, small‐ and intermediate‐sized herbivores may therefore be forced to forage in patches of low‐quality food (Hernández & Laundré, 2005) instead of foraging in patches of high‐quality food, because they have less time to search for the scarce and small patches of high‐quality food (Shipley, 2007), or because traveling between small patches of high‐quality food increases the probability of detection by predators (Broom & Ruxton, 2005; Eccard & Liesenjohann, 2014). Moreover, patches that contain low‐quality food (often tall vegetation) offer more cover for prey at risk that hides from predators (Riginos & Grace, 2008). If predators force small‐ and intermediate‐sized herbivores to seek cover in patches of low‐quality food, then these herbivores must spend more time foraging, because of the increased search and handling times, than in patches of high‐quality food (Heuermann, Van Langevelde, Van Wieren, & Prins, 2011).

Foraging of herbivores can also be negatively affected by the presence of competitors (Ferretti et al., 2015; Focardi, Aragno, Montanaro, & Riga, 2006). Similarity in body mass and morphology is expected to increase competition, whereas differences in body mass and morphology allow habitat segregation between herbivores (Prins & Olff, 1998). For example, the bite size of smaller‐sized herbivores allows proportionally higher intakes of high‐quality food on grasslands that contained a lower quantity of food relative to larger‐sized herbivores (Wilmshurst, Fryxell, & Bergman, 2000). Thus, intermediate‐sized herbivores can be excluded by smaller herbivores if densities of smaller competitors are high, plant biomass is low, and food becomes depleted and unavailable (Shipley, 2007). For intermediate‐sized herbivores, locations with high‐quality food are then hypothesized to be traded for locations with low‐quality food during times of high competitor activity by smaller herbivores.

While the importance of predation risk (Lima & Dill, 1990), competition (Arsenault & Owen‐Smith, 2002; Prins & Olff, 1998), and forage quality and quantity (Barboza, Parker, & Hume, 2009) for foraging time has been recognized widely, their relative importance in determining where and what intermediate‐sized herbivores forage remains unclear (Arsenault & Owen‐Smith, 2002; Brown & Kotler, 2004; Morris, 2009). Whereas many studies focus on the trade‐off between resource acquisition and predation risk (Laundré, 2010; Sih, 2005; Thaker et al., 2011), few studies simultaneously consider the trade‐off with competition (Lima, 1998; Morris, 2002, 2009). It has been hypothesized that the effect of intra‐ and interspecific competition on foraging behavior could be more important than the effect of predation risk (Grand & Dill, 1999a; Halliday & Morris, 2013), especially if resource availability is low (Chesson & Kuang, 2008), and herbivores are similar‐sized (Sinclair, 1985). Our objective was therefore to test the relative importance of predator and competitor activity, and forage quality and quantity on the proportion of time spent in a vegetation type and the proportion of time spent foraging by the intermediate‐sized herbivore European hare (*Lepus europaeus*). We focused on red fox (*Vulpes vulpes*) as the main predator of European hare and European rabbit (*Oryctolagus cuniculus*) as the main competitor of European hare. Red fox can substantially impact hare populations as a predator (Knauer, Küchenhoff, & Pilz, 2010; Schmidt, Asferg, & Forchhammer, 2004). European hares and rabbits have a substantial overlap in resources (Kuijper, van Wieren, & Bakker, 2004) and are classified as trophic competitors when sympatric (Homolka, 1987). Rabbits are central‐place foragers that are smaller than hares and more ecologically specialized, and thus, we expect rabbits to outcompete hares (Shipley, 2007). Additionally, rabbit activity is positively related to the amount of foraging bouts away from its burrow (Bakker, Reiffers, Olff, & Gleichman, 2005).

As argued earlier, we expected that, during times that predators and smaller competitors are more active, intermediate‐sized herbivores spent more time in vegetation types that contain lower food quality (Prins & Olff, 1998; Shipley, 2007; Wilmshurst et al., 2000), and therefore, they must spend more time on foraging. Whereas during times that predators or smaller competitors are less active, intermediate‐sized herbivores spend more time in vegetation types that contain higher food quality. and therefore, they could spend less time on foraging. We hypothesized that, if resource levels are high, time spent foraging by intermediate‐sized herbivores is more strongly affected by predator activity than by competitor activity (Chesson & Kuang, 2008; Grand & Dill, 1999a), forage quality, or forage quantity (Hopcraft et al., 2010; Sinclair et al., 2003).

## 2 MATERIALS AND METHODS

We conducted the study in the coastal‐dune landscape “Noordhollands Duinreservaat” near Castricum (52°33′N, 4°38′E) in the Netherlands. Three areas, Castricum (ICAS) (325 ha), Vennewater (VW) (275 ha), and Koningsbos (KB) (50 ha), were selected based on previous sightings of hare. The coastal‐dune landscape on nutrient‐poor sandy soils contained a mosaic of 20 dune vegetation types relevant for hares (Appendix, Table A1). However, the overall resource availability for vegetation types is high because of atmospheric deposition (Kooijman, Dopheide, Sevink, Takken, & Verstraten, 1998). Red fox was present at a high density of 5 ind/km^2^, whereas European rabbit, was present at a low density of 2 ind/km transect (Mulder, 2005). We have no independent estimate of hare density in the area. However, we assessed the coastal‐dune landscape to be good hare habitat (±15 hares/km^2^), with no hunting, but high predator density.

### 2.1 Hare foraging behavior and location

To measure the time spent foraging, we GPS tracked 12 hares in the study area between 15 October 2014 and the first of January 2015. During this period, female hares store energy, because they are capital breeders, especially when having their first litter (Valencak, Tataruch, & Ruf, 2009). We therefore expected female hares to be more selective in their foraging behavior, even more because the nutrient quality of the vegetation during the study period is relatively low (Smith, Jennings, & Harris, 2005).

Hares were flushed by a line of beaters and caught using Speedset static hare nets (height 45 cm, with 13 cm full mesh; JB's Nets, Alexandria, UK). Caught hares were quickly removed from the nets, blindfolded (Paci, Ferretti, & Bagliacca, 2012), and temporarily kept in darkened wooden boxes to reduce stress. Healthy hares were tagged without sedation (Gerritsmann, Stalder, Seilern‐Moy, Knauer, & Walzer, 2012) immediately after all hares in an area were flushed. Hares were equipped with a neck belt that contained a GPS and an accelerometer (69 g, 1.8 ± 0.2% of body weight) with wireless communication (Type A, E‐obs GmBH, Gruenwald, Germany). After tagging, we measured body weight $(\overset{¯}{X}$ ± *SD*, 3,719 ± 281 g, and determined sex (7 females, 4 males, 1 unknown) and age (6 individuals < 1 year old, 5 > 1 year, 1 unknown) of the hares. The capturing of hares was executed under the approval of the Wageningen University Animal Experiment Committee (no. 2014034.b) and followed the EU Directive 2010/63 on the protection of animals used for scientific purposes.

Hares were allowed to settle down for a period of 5 days after capturing before the GPS and accelerometer started recording data (Petrovan, Ward, & Wheeler, 2013). The GPS position of individual hares was recorded every 12 min, 24 hr a day. Acceleration in three axes was recorded every minute for 8‐s, 24 hr a day, with a frequency of 31.62 Hz, allowing detailed determination of behavior. The raw data of accelerometer recordings were transformed into physical units (m/s^2^) by the following:

(1) $$a_{i}\;=\;\left(n_{i}\;-\;n_{i,\mathrm{zerog}}\right)\cdot c_{i}\cdot g$$

where *a*_*i*_ (m/s^2^) is the acceleration of axis *i*,* n*_*i*_ is the raw data (unitless values) of one axis, *n*_*i*,zerog_ is the raw data without gravitational force and no dynamic acceleration (unitless value), *c*_*i*_ is a constant (unitless value), and *g* is the acceleration caused by earth gravitation (9.81 m/s^2^). The constants *c*_*i*_ and *n*_*i*,zerog_ of each accelerometer were calibrated and measured before the start of the study following E‐obs protocol (http://www.e-obs.de).

For each 1‐s segment of acceleration, we calculated the following parameters for each hare (Bom, Bouten, Piersma, Oosterbeek, & Van Gils, 2014; Nathan et al., 2012):

1. For each axis separately: (a) standard deviation of the static acceleration, (b) maximum dynamic acceleration component, (c) arithmetic mean of the smoothed time series (moving median with window size k = 5), (d) skewness, and (e) kurtosis.
2. For all three axes combined: (a) the resultant of the *x*‐, *y*‐ and *z*‐axis of the parameters described at (1), as the square root of the sum‐of‐squares of the three axes, (b) dynamic body acceleration, and (c) overall dynamic body acceleration (ODBA).

To label the accelerometer data with behaviors, we recorded 8,771 s of behavior (range: 3–4,122 s, *n* = 8, 4 females, 2 males, and 2 unknown) using a handheld video of tagged hares in coastal‐dune landscapes. Video fragments were labeled with one of 8 types of behavior (laying, sitting, sitting alert, grooming, scratching, chewing, foraging, and moving) using the software Avidemux (2.6.6). Only 1‐s segments that contained 100% of the same behavior were used in the subsequent analysis. Decision tree software (AcceleRater, Resheff, Rotics, Harel, Spiegel, & Nathan, 2014) together with the labeled accelerometer segments were used to classify the unlabeled accelerometer data into foraging (precision: 83%, accuracy: 92%, recall: 93%).

### 2.2 Forage quality and quantity

We used a high‐resolution GIS map (1:5.000) of vegetation types in the study area (Everts, Pranger, Tolman, & De Vries, 2008, 2009) to extract the vegetation types for the corresponding GPS locations of hares. Forage quality and quantity were estimated in the vegetation types that were used by the tracked hares. We measured quantity (edible biomass) and quality (concentration of nutrients) of the vegetation as forage for the hares in the vegetation types based on a selection of the locally most important plant species in the diet of hares, namely *Festuca rubra*,* Agrostis capillaris*,* Poa pratensis*,* Holcus lanatus*,* Poa trivialis*,* Taraxacum officinale*,* Rubus caesisus* (Kuijper, Beek, Van Wieren, & Bakker, 2008; professional judgement S. E. van Wieren) and a commercial flower bulb species.

For each plant species, we hand‐plucked mixed samples of edible biomass, that is, green plant parts that have a high nutritional value and are selected by hares (Homolka, 1987), in six randomly placed circular plots (10 m radius) in each vegetation type. To assess whether forage quality and edible biomass varied over the study period, we collected these mixed samples in two sample sessions (Oct & Jan). In each vegetation type, we visually estimated the percentage cover of each plant species in six 2 × 2 m quadrants (using 40 × 40 cm subquadrants of the 2 × 2 m quadrants) and measured their average height at five orthogonal locations. We assumed plant parts at more than 50 cm from ground level were unavailable as forage for the hares. For each plant species, we estimated the conversion factor between the total biomass, edible biomass. and the volume of the plant species by removing all vegetation in two 50 × 50 cm quadrants (i.e., one with the highest and one with the lowest average height of the plant species) located inside the six 2 × 2 m quadrants.

Plant parts were air‐dried, stored, and chemically analyzed for the percentage of N, P, Ca, and NDF (neutral detergent fiber) in the biomass. Because the amount of fiber in the vegetation can reduce food intake and affect foraging behavior, especially for small herbivores that generally avoid vegetation types with high fiber content, we measured NDF as an index of plant fiber content (i.e., total cell walls) (Barboza et al., 2009). We did not find any changes in the forage quality and edible biomass of the vegetation types between the two sample sessions (October & January). For each nutrient, average concentration of each vegetation type was calculated by averaging the percentage of nutrients for each plant species present in the vegetation type, weighted by their volume per square meter up to 50 cm in height. We calculated the average edible biomass (g/m^2^) for each vegetation type by summing the amount of edible biomass (g) of all plant species in one square meter of the vegetation type up to 50 cm in height.

The average nutrient and plant fiber concentrations of the vegetation types were highly correlated (Appendix, Table A1). We therefore extracted two PCA axes of the nutrients (% of N, P, Ca) and the fiber content (% of NDF) by a principal component analysis (SPSS version 23.0). Axes were rotated by a Varimax with Kaiser Normalization. Factor scores above 1 (Kaiser, 1960) were calculated and standardized by the Anderson–Rubin method (DiStefano, Zhu, & Mîndrilă, 2009), which ensures orthogonality of the estimated factors. The first PCA axis was strongly positively correlated with the percentage of N and P in the edible biomass of the vegetation (Table 1). The second PCA axis was strongly positively correlated with the percentage of NDF and strongly negatively correlated with the percentage Ca in the edible biomass of the vegetation. We multiplied the 2nd PCA axis by −1 to get a consistent interpretation of forage quality, because we associated poor forage quality with a higher percentage of NDF.

**Table 1:** Rotated PCA component coefficient values of forage quality of the vegetation types in the coastal‐dune landscape (*n* = 20). Note the multiplication of PCA axis 2 with −1 to get a consistent interpretation of forage quality (i.e., QL2)

Nutrients and NDF	Forage quality^a^ (% nutrients in edible biomass)	
	QL1 = PCA axis 169.7% (2.8)^b^	QL2 = −1 × PCA axis 2 27.7% (1.1)
N	**0.96**	−0.13
P	**0.96**	−0.21
Ca	−0.55	**0.83**
NDF	0.04	−**1.00**

Notes

NDF: neutral detergent fiber on ash‐in‐basis.

^a^Varimax with Kaiser Normalization; listwise deletion, PCA components >0.6 are bold; ^b^Percentage of variance explained by component (eigenvalue of component).

### 2.3 Predator & competitor activity

We investigated predator and competitor activity using a network of camera traps. Camera traps locations were based on accessibility, expected use of vegetation types, preferred plant species by hare, and covered 13 vegetation types in the same areas that were used by the tracked hares. Forty‐two camera traps (Reconyx Hyperfire: HC500 and HC600, infrared trigger) were randomly placed in open and half‐open vegetation at a height of 30 cm for about 5 sessions of 15 days between 16 October 2014 and 8 January 2015 (208 camera locations). Open vegetation structure has often a high forage quality for hares (Kuijper et al., 2008) where they can easily spot predators, whereas half‐open vegetation structures provide lower forage quality, but visual cover (Neumann, Schai‐Braun, Weber, & Amrhein, 2011). Camera traps were interspaced on average by 689 m (*SD* ± 1,189, *n* = 135), >25 m from waterbodies and >16 m from recreational paths, and set up according to the protocol of Jansen, Forrester, and McShea (2014).

Camera traps were configured to record a burst of ten photographs when triggered, without any time lapse between bursts. Visits were visually assessed from sequences of photographs and were counted as a new visit if the quiet period in the beginning was longer than 120 s. Overall predator and competitor activity was quantified as the total number of camera visits by predators or competitors in the study area during a day.

### 2.4 Data analysis

We investigated the effects of predator and competitor activity and forage quality and edible biomass with their interactions on (a) the proportion of GPS fixes in a vegetation type and on (b) the proportion of time spent foraging in a vegetation type, on a per day basis. We ran multiple generalized linear mixed models in R (glmer, package lme4 version 1.1‐13) for both analyses, with a binomial error structure and logit link. The total number of GPS fixes on a day and the total number of seconds of measured hare foraging time spent recorded on a day were set as the upper limit of the binomial structure. We used predator activity, competitor activity, forage quality, edible biomass, and vegetation height as predictor variables. We included the average vegetation height as an indicator for prey cover (Verdolin, 2006). Forage quality and vegetation height are often interpreted to be inversely related to each other (see, e.g., Bell, 1971). In our study, however, forage quality was measured in the edible biomass only, up to 50 cm of height. Nevertheless, plant fiber concentration (2nd PCA component) and vegetation height were moderately correlated (*r* = −0.58, *p* < 0.01, *n* = 20), whereas plant nutrient concentration (1st PCA component) and vegetation height were not correlated (*r* = −0.12, *p* = 0.62, *n* = 20). Predator and competitor activity, edible biomass, and vegetation height were standardized and scaled by dividing their mean by two standard deviations (Gelman, 2008). Multicollinearity of continuous predictor variables was assessed (Zuur, Ieno, & Elphick, 2010). The Variance Inflation Factor (VIF) of all continuous predictor variables remained below 2.1 in both analyses.

Candidate models were used to assess the relative strength of our hypotheses following Grueber, Nakagawa, Laws, and Jamieson (2011). We generated 24 candidate models from the combinations of the predictor variables, including an intercept‐only model.

Candidate models to explain the proportion of GPS fixes in a vegetation type (Appendix, Table A2) included date as random factor. Date was also used as the repeated measurement variable for each vegetation type. There was no autocorrelation between dates (first 5 days : $\overset{¯}{X}$ ± *SD, r *=* *0.15 ± 0.13). All candidate models included area size of the vegetation type as control variable. We excluded vegetation types for which we had no data on forage quality and edible biomass, and we excluded records when there was no activity of predators or competitors to create a dataset without missing values, for which candidate models could be compared by the small sample Akaike information criterion (AICc).

Candidate models to explain the proportion of time spent foraging in a vegetation type (Appendix, Table A3) included area, date, and hare‐ID in a specific vegetation type as random factors. Hare‐ID in a specific vegetation type was nested within date that was nested within area. Date was used as the repeated measurement variable. There was no autocorrelation between dates (first 5 days: $\overset{¯}{X}$ ± *SD, r *=* *−0.04 ± 0.04). All candidate models included area type and the sex of the animals as control variables. Body weight did not improve the fit of the models and was left out as a control variable. We excluded the hare of unknown sex to create a dataset without missing values, for which candidate models could be compared by AICc.

We assessed the relative weights of parsimonious models only, that is, we preferred nested models that could explain the data with as few predictor variables as possible. We thus removed the complex models with higher values of AICc that had more predictor variables than the nested (parsimonious) ones. We then performed full‐model averaging of all the parsimonious models to estimate the beta coefficients (*β*) and the (conditional) average standard errors ($\hat{{\mathrm{SE}}_{\beta }}$) of model parameters. Overdispersion of models was assessed by the Pearson's chi‐square over the residual degrees of freedom of the model (Crawley, 2007). Assumptions were verified by visual inspection of residuals plotted against the predicted (full model), and outliers were identified with Cook's Distance.

## 3 RESULTS

We found that the proportion of time that hares spent in a vegetation type was best explained by the model that included the interaction between fox activity and forage quality (2nd PCA component), the interaction between fox activity and edible biomass, and the interaction between fox activity and vegetation height (Appendix, Table A2). The top model had a total relative weight of 87% and thus had the best fit to our data. Models that included rabbit activity or the first PCA component of forage quality (N and P) received very low relative model weights.

**Figure 1:**
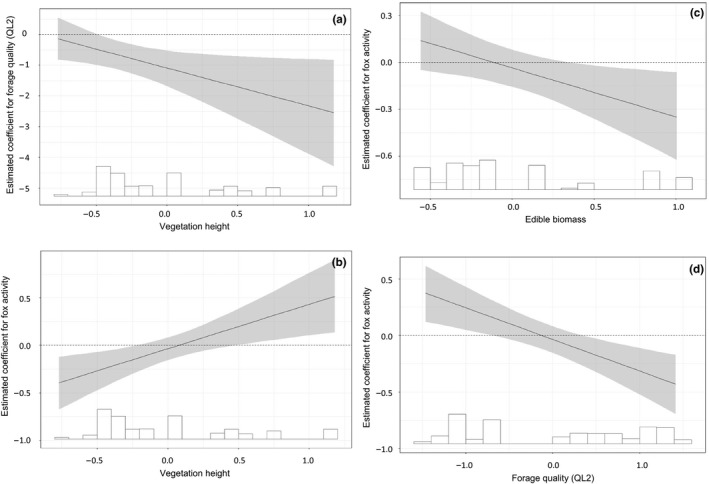
a‐d: The estimated beta (*β*) coefficient ($\overset{¯}{X}$ ± 95% CI) between the proportion of GPS fixes of European hares in a vegetation type and (a) forage quality (NDF(−) and Ca(+)) by vegetation height (cm), (b) fox activity by vegetation height (cm), (c) fox activity by edible biomass, and (d) fox activity by forage quality (NDF(−) and Ca(+)). Histogram shows distribution of the conditional coefficient

Forage quality (2nd PCA component) was negatively correlated with the proportion of time spent in a vegetation type (Table 2). Hares spent less time in vegetation types that contained a higher percentage of Ca, whereas hares spent more time in vegetation types that contained a higher percentage of NDF. This effect became stronger with increasing vegetation heights (Figure 1a). The coefficient of fox activity on the proportion of time spent in a vegetation type was positively related to vegetation height (Figure 1b), but negatively related to edible biomass (Figure 1c) and forage quality (2nd PCA component; Figure 1d). During days that foxes were more active, hares thus spent a higher proportion of time in tall vegetation types and vegetation types that contained a relatively low edible biomass and quality of forage. We found no interaction between rabbit activity and forage quality or between rabbit activity and edible biomass on the proportion of time spent in a vegetation type. The standardized beta coefficients show that forage quality or edible biomass more strongly affected the proportion of time hares spent in a vegetation type than the activity of foxes (Table 2).

**Table 2:** Results of full‐model conditional averaging of all parsimonious generalized linear mixed models on the effect of predator activity and its interaction with forage quality, edible biomass, and vegetation height on the proportion of GPS fixes of European hares in a vegetation type

Variables	Estimate (*β*)^a^	Conditional $\hat{{\mathrm{SE}}_{\beta }}$	Z value	2.5%–97.5% C.I.	Effect^b^	*W*_p_
Intercept	−3.77	0.24	15.8	−4.24 to −3.30	***	1.00
EB	0.58	0.43	1.3	−0.27 to 1.43		0.88
VH	0.88	0.63	1.4	−0.36 to 2.12		0.96
QL2	−0.72	0.28	2.5	−1.27 to −0.16	*	0.90
QL2*VH	−1.24	0.58	2.1	−2.37 to −0.10	*	0.08
Fox	−0.03	0.06	0.6	−0.15 to 0.08		0.90
Fox*EB	−0.31	0.13	2.4	−0.57 to −0.06	*	0.88
Fox*VH	0.47	0.16	2.9	0.15 to 0.78	**	0.88
Fox*QL2	−0.28	0.09	3.3	−0.44 to −0.11	**	0.90
Area size	1.73	0.44	4.0	0.88 to 2.59	***	1.00

Notes

EB: edible biomass (g/m^2^); VH: vegetation height (cm); QL2: −1*2nd PCA component of forage quality: NDF (−) and Ca (+); fox: red fox activity (log); area size: area size of vegetation types (log); *W*p: Akaike predictor weight.

^a^Beta coefficients standardized by 2*SD (Gelman, 2008). Beta of interaction is difference in slope between the two values when the covariate increases 1 standard deviation; ^b^Effect = 95% confidence interval does not include zero. *p < 0.05, **p < 0.01, ***p < 0.001. Models are based on 979 observations of 11 hare in 20 vegetation types over 71 days.

The proportion of time hares spent foraging in a vegetation type was best explained by the model that included the interaction between fox activity and forage quality (2nd PCA component; Appendix, Table A3). The top model was closely followed by a similar model that contained rabbit activity instead of fox activity. The top two models had a total relative weight of 91% and thus had the best fit to our data. Models that included the first PCA component of forage quality (N and P) received lower relative model weights (≤0.01) in the model set.

**Figure 2:**
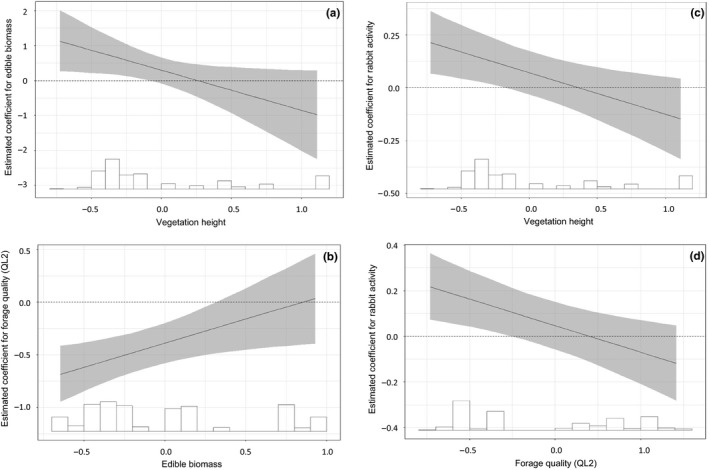
a‐d: The estimated beta (*β*) coefficient ($\overset{¯}{X}$ ± 95% CI) between the proportion of time spent foraging by European hares in a vegetation type and (a) edible biomass by vegetation height (cm), (b) forage quality (NDF(−) and Ca(+)) by edible biomass, (c) rabbit activity by vegetation height (cm), and (d) rabbit activity by forage quality (NDF(−) and Ca(+)). Histogram shows distribution of the conditional coefficient

Vegetation height and forage quality (2nd PCA component) were on average negatively correlated with the proportion of time spent foraging; however, fox activity was positively correlated with the proportion of time spent foraging (Table 3). Hare thus spent a higher proportion of time foraging in short vegetation types and in vegetation types with a lower percentage of Ca and a higher percentage of NDF. They also spent a higher proportion of time foraging during days that foxes were more active. In tall vegetation, edible biomass was negatively related to the proportion of time spent foraging (Figure 2a), whereas in short vegetation, edible biomass was positively related to the proportion of time spent foraging (Figure 2a). In vegetation types with more edible biomass, forage quality (2nd PCA component) was less negatively related to the proportion of time spent foraging by hares (Figure 2b). The effect of rabbit activity on the proportion of time spent foraging in a vegetation type was negatively related to vegetation height (Figure 2c) and forage quality (2nd PCA component) (Figure 2d). During days that rabbits were more active, hares thus spent a higher proportion of time foraging in short vegetation types and in vegetation types that contained a relatively low quality of forage. Additionally, males spent a lower proportion of time foraging than females. The standardized beta coefficients show that forage quality, edible biomass, and vegetation height more strongly affected the proportion of time hares spent foraging in a vegetation type than the activity of foxes or rabbits.

**Table 3:** Results of full‐model conditional averaging of all parsimonious generalized linear mixed models on the effect of predator and competitor activity and its interaction with forage quality, edible biomass, and vegetation height on the proportion of time spent foraging of European hares in a vegetation type

Variables	Estimate (*β*)^a^	Conditional $\hat{{\mathrm{SE}}_{\beta }}$	Z value	2.5% to 97.5% C.I.	Effect^b^	*W*_p_
Intercept	−0.71	0.13	5.3	−0.97 to −0.45	***	1.00
EB	0.09	0.19	0.5	−0.28 to 0.46		0.05
VH	−0.58	0.20	2.9	−0.97 to −0.19	**	0.01
EB*VH	−1.14	0.57	2.0	−2.25 to −0.02	*	<0.01
QL1	0.19	0.13	1.5	−0.06 to 0.45		<0.01
QL2	−0.43	0.10	4.3	−0.62 to −0.23	***	0.99
QL2*EB	0.46	0.20	2.3	0.07 to 0.85	*	0.05
Fox	0.14	0.05	2.7	0.04 to 0.23	**	0.65
Fox*VH	−0.09	0.07	1.2	−0.24 to 0.06		<0.01
Fox*QL1	−0.09	0.06	1.6	−0.21 to 0.02		<0.01
Fox*QL2	−0.06	0.04	1.4	−0.14 to 0.02		0.64
Rabbit	0.05	0.05	0.9	−0.06 to 0.15		0.27
Rabbit*VH	−0.19	0.08	2.5	−0.34 to −0.04	*	<0.01
Rabbit*QL2	−0.12	0.04	2.7	−0.20 to −0.03	**	0.27
Sex^c^	−0.48	0.20	2.4	−0.87 to −0.09	*	1.00
Area type^d^	−0.25	0.19	1.3	−0.63 to 0.13		1.00

Notes

EB: edible biomass (g/m^2^); VH: vegetation height (cm); QL1: 1st PCA component of forage quality: N and P; QL2: −1*2nd PCA component of forage quality: NDF (−) and Ca (+); fox: red fox activity (log); rabbit: rabbit activity; *W*p: Akaike predictor weight.

^a^Beta coefficients standardized by 2*SD (Gelman, 2008). Beta of interaction is difference in slope between the two values when the covariate increases 1 standard deviation; ^b^Effect = 95% confidence interval does not include zero. *p < 0.05, **p < 0.01, ***p < 0.001. Models are based on 2,843 observations of 11 hare in 19 vegetation types in 2 areas over 79 days; ^c^Reference category for sex is female; ^d^Reference category for area type is Vennewater.

## 4 DISCUSSION

We have tested the relative importance of predator and competitor activity, and forage quality and quantity on the proportion of time spent in a vegetation type and the proportion of time spent foraging by the intermediate‐sized herbivore European hare. Most studies (>75%) that investigate the trade‐off between foraging behavior and predation risk using giving‐up density focus on small central‐place foragers (i.e., rodents <1 kg) (Verdolin, 2006; e.g., squirrels, mice, and voles). Very few studies focus on intermediate‐sized (<20 kg) free‐ranging herbivores (but see. e.g., Hodges & Sinclair, 2005; Shrader, Kerley, Brown, & Kotler, 2012; Crowell et al., 2016), which use a different foraging strategy, and show a different response toward predators and competitors (Potts, Harris, & Giuggioli, 2012; Shrader et al., 2012). Moreover, studies that focus on giving‐up density are limited by the artificiality of the food patches, especially the quality of the food offered, and the predictability of the food patch (Bedoya‐Perez, Carthey, Mella, McArthur, & Banks, 2013).

Our first expectation was that when predators and smaller competitors were more active, intermediate‐sized herbivores spent more time in vegetation types that contained a lower food quality. We found that increased activity by smaller competitors did not affect the proportion of time hares spent in a certain vegetation type. However, during increased activity of predators, hares spent a higher proportion of time in vegetation types that had tall vegetation or a low food quality or quantity. In this study, food quality and vegetation height were measured separately and were not (N and P concentration) to moderately (Ca and NDF concentration) correlated with vegetation height. Therefore, we interpreted vegetation height as an indicator for prey cover only. Tall structure‐rich vegetation provides cover and protection for prey that hides from predators (Verdolin, 2006) and is used as resting place by hares during the day (Neumann et al., 2011). Besides, hares make use of cryptic coloration in tall vegetation to evade predators (Focardi & Rizzotto, 1999). Unlike European hares, snowshoe hares (Hodges & Sinclair, 2005) and roe deer (Samelius, Andrén, Kjellander, & Liberg, 2013) did not spent more time in low‐risk vegetation types to reduce predation risk, possibly because of differences in predator type or prey escape mode (Wirsing, Cameron, & Heithaus, 2010). Even though hares use flight in short vegetation to escape predators (Focardi & Rizzotto, 1999), we found that high fox activity negatively affected the proportion of time that hares spent in short vegetation. The reason that hare does not spend more time in short vegetation during times of high risk is probably that hares cannot detect foxes early enough or escape from these foxes if patches of short vegetation are smaller than their minimum flight distance. Prey escape mode (Wirsing et al., 2010) and landscape features (Heithaus, Wirsing, Burkholder, Thomson, & Dill, 2009), such as the small size of patches or patch distribution, may thus favor fox hunting in patches of short vegetation (Kauffman et al., 2007; Weterings et al., 2016). It seems that habitat shifts as a result of the antipredator behavior of hare is context dependent (Kuijper, Bubnicki, Churski, Mols, & Van Hooft, 2015), namely that it depends on the patch size of the vegetation.

Independent of predator activity, hares also spent more time in vegetation types that contained a low food quality. Especially in winter, hare can forage on grasses that contain a higher concentration of fibers with lower levels of lignin compared to dicotyledonous species (Iason & Van Wieren, 1999).

Our second expectation was that if intermediate‐sized herbivores had to spend more time in vegetation types that contained a lower food quality they also had to spend more time on foraging. As expected, our hares spent a higher proportion of time spent foraging in vegetation types with a higher concentration of fibers (i.e., NDF) in the edible biomass, especially in vegetation types with a low edible biomass. As plant bite sizes are correlated with biomass, smaller bites in vegetation types with a lower edible biomass require more handling time and will thus reduce forage intake (Shipley, 2007) and increase foraging time (Heuermann et al., 2011). Additionally, the concentration of fibers in the food will negatively affect forage intake, although this strongly depends on the type of herbivore digestion system (Bell, 1971). In vegetation types with a higher concentration of fibers, hares, which have a relatively short digestion system, maximize the passage rate of forage (Stott, 2007), and thus spent more time to foraging. Remarkably, independent of the vegetation type (i.e., low and high risk), the proportion of time spent foraging also increased when foxes were more active (see Table 3). This implies that hares not only perceived a predation risk that was nonuniformly spread over the landscape (i.e., low‐ and high‐risk vegetation types) (Kotler & Blaustein, 1995), but hares also perceived a predation risk that was uniformly spread over the landscape. At a higher predation risk, free‐ranging herbivores increase their time spent foraging if they have no safe refuges from predators, especially if “predator and prey are of similar body size and locomotion” (Eccard, Pusenius, Sundell, Halle, & Ylönen, 2008, p.726), like the European hare and the red fox.

Besides that hare foraging behavior was affected by fox activity, hares spent a higher proportion of time foraging when rabbits were more active, especially in vegetation types with a high concentration of fibers and short vegetation types. First, spending time in vegetation types with a high concentration of fibers would allow the larger hare to avoid competition with the smaller rabbit (given that larger herbivores have the ability to tolerate low forage quality; Bell, 1971), but increased the proportion of foraging time. Second, rabbits can dilute predation risk for hares in the risky short vegetation types, particularly, because the smaller rabbits are the stronger competitor (see Shipley, 2007) and experience a higher individual mortality risk by predation than hares (Grand & Dill, 1999b). Because of this, hares are expected to aggregate with rabbits in the “risky but productive” short vegetation type (Grand & Dill, 1999b). This would mean that when rabbits and hares are active in short vegetation, hare would select plants with a higher fiber concentration, while rabbits would select plants with a lower fiber concentration.

Our results show that food quality and quantity more strongly affected hare foraging behavior than the activity of predators, whereas the activity of smaller competitors was least important. Predation risk might be less strong than the effect of resource acquisition, probably because the relative size difference between our prey species and its predator was small (Sinclair et al., 2003). The range in nutrient concentrations measured in the edible biomass (Appendix, Table A1) seems to reflect the natural variability in coastal‐dune landscapes (e.g., see % of NDF in Lamoot, 2004). The absence of intraspecific‐group competition (Grand & Dill, 1999a), and the low density of smaller competitors (Hopcraft et al., 2010) in the coastal‐dune landscape, possibly marginalized the effects of small competitor activity on hare foraging time. Additionally, predation risk is stronger than competition in the landscapes of high resource availability (Chesson & Kuang, 2008) that are present in the Dutch dune‐coastal landscape (Kooijman et al., 1998), were Calcium is not a limiting resource (Barboza et al., 2009).

By investigating the relative importance of factors that affect behavioral trade‐offs in complex landscapes, we can get insight into the mechanisms that determine spatial distribution of herbivores. Although predation risk affected space use and foraging behavior, and competition affected foraging behavior, it seems that we need to reconsider the relative importance of the landscape of food in a world of fear and competition.

## CONFLICT OF INTEREST

The authors declare to have no conflict of interest.

## AUTHOR CONTRIBUTIONS

MW, SM, and SvW conceived and designed the study. MW and SM contributed to acquisition of data. MW, HP, and FvL analyzed and interpreted the data. MW, SM, HP, FvL, and SvW wrote the manuscript. All authors read, reviewed, and approved the final manuscript.

## DATA ACCESSIBILITY

Data available from the Dryad Digital Repository: https://doi.org/10.5061/dryad.95r0850

## APPENDIX 1

### 1.1

**Table A1:** Characteristics of the vegetation types in the coastal‐dune landscape “Noordhollands Duinreservaat” near Castricum, the Netherlands

Vegetation type	Area size (ha)	# hares foraging	# camera locations	# plots	Average percentage of GPS fixes/day of hares (±*SD*)	Plant species in hare diet^a^	Average percentage of time spent foraging (±*SD*)	Biomass (g/m^2^)*	Edible biomass (g/m^2^)^b^	Nutrients in the vegetation (%)				Forage quality	
										N	P	Ca	NDF	QL1	QL2
Agriculture	40.8	10	‐	6	21.3 ± 9.7	2, 3, 4, 5, 6	56.0 ± 12.3	105	40	3.7	0.4	0.5	48	0.2	0.6
Flower‐rich grasslands	43.3	6	24	5	9.0 ± 7.4	1, 2, 4, 6	43.1 ± 22.0	148	58	3.6	0.4	0.5	51	−0.2	1.1
Bulb fields	1.3	2	‐	1	1.2 ± 1.5	8	37.3 ± 19.3	642	210	5.1	0.7	0.3	39	3.3	−0.9
Dune grasslands	74.0	11	59	6	11.8 ± 6.4	1, 2, 3, 5, 6, 7	41.3 ± 23.2	242	110	3.6	0.3	1.1	41	0.0	−0.6
Burnet rose, creeping willow‐, blackberry thicket	37.0	8	29	6	5.9 ± 4.9	1, 2, 3, 4, 6, 7	30.8 ± 23.9	418	227	4.0	0.4	0.9	43	0.5	−0.2
Bare sand	3.6	5	‐	7	0.8 ± 0.7	1, 2, 3, 7	36.2 ± 16.2	12	6	3.7	0.3	0.6	48	−0.1	0.7
Calcareous dune grassland	125.2	11	74	5	14.7 ± 7.9	1, 2, 3, 4, 7	36.2 ± 24.2	467	207	3.3	0.3	0.5	51	−0.6	1.2
Calcareous dune valleys	2.9	1	2	5	1.0 ± 1.5	1, 2, 3, 4, 5, 6	57.4 ± 32.7	243	138	4.1	0.4	0.9	42	0.7	−0.3
Deciduous forest	65.1	8	4	3	2.3 ± 2.8	2, 4, 5, 7	29.4 ± 30.5	10	4	4.1	0.5	0.6	45	1.1	−0.1
Coniferous forest	37.0	6	‐	1	0.5 ± 1.2	7	20.3 ± 15.2	2	0	2.6	0.2	1.6	35	−1.3	−1.5
Former agriculture	7.5	2	‐	3	7.2 ± 3.4	1, 2, 4, 7	22.2 ± 15.1	153	65	3.7	0.4	0.5	48	0.2	0.7
Other	6.7	3	‐	2	0.8 ± 1.2	2	45.5 ± 28.6	1	0	3.6	0.3	0.4	53	−0.4	1.4
Other forests	18.5	7	1	4	2.5 ± 2.1	2, 3, 5, 7	32.3 ± 24.3	256	52	2.9	0.3	1.2	41	−1.0	−0.5
Reed swamp	17.4	3	1	2	4.3 ± 6.6	4, 5, 7	52.1 ± 27.5	429	39	2.8	0.2	1.5	36	−1.1	−1.3
Reed swamp communities	0.7	4	1	2	0.5 ± 0.7	1, 2, 3, 4, 5, 6	33.0 ± 25.4	79	31	3.7	0.4	0.5	49	0.2	0.8
Herbaceous, fault, and mantle communities	3.7	8	‐	4	1.1 ± 1.5	2, 4, 5, 7	35.3 ± 26.0	81	9	2.9	0.3	1.4	38	−0.8	−1.2
Thickets	75.6	11	6	4	8.2 ± 4.4	1, 2, 4, 5, 7	27.9 ± 24.3	360	97	3.1	0.3	1.4	37	−0.7	−1.1
Nutrient‐rich grasslands	20.8	9	2	5	8.1 ± 7.4	1, 2, 3, 4, 5, 6	49.0 ± 24.1	88	32	3.5	0.4	0.4	51	−0.1	1.1
Nutrient‐rich pioneer communities, flood meadows, and pace vegetation	1.9	4	1	6	1.8 ± 2.5	2, 3, 4, 5	31.9 ± 23.1	67	25	3.6	0.4	0.4	53	−0.3	1.3
Near‐shore communities	13.5	3	4	6	0.7 ± 0.9	1, 2, 3, 4, 7	30.1 ± 22.5	267	145	4.1	0.3	1.2	37	0.7	−1.1

Notes

NDF: neutral detergent fiber on ash‐in‐basis; QL1: 1st PCA component nutrient quality of the vegetation; QL2: 2nd PCA component nutrient quality of the vegetation.

^a^Plant species are ordered by fiber concentration from high to low: 1 = Festuca rubra, 2 = Agrostis capillaris, 3 = Poa pratensis, 4 = Holcus lanatus, 5 = Poa trivialis, 6 = Taraxacum officinale, 7 = Rubus caesisus, and 8 = commercial bulb species; ^b^(edible) biomass is calculated up to a height of 50 cm.

**Table A2:** Results of the generalized linear mixed model on the effect of predator and competitor activity and its interaction with forage quality, edible biomass, and vegetation height on the proportion of GPS fixes of European hares in a vegetation type

Rank	Model type	*df*	AICc	ΔAIC	*W*_*i*_ a
1	EB + VH + QL2 + fox + fox*EB + fox*VH + fox*QL2	14	8,672.4	0.0	0.87
2	VH + QL2 + QL2*VH	10	8,677.2	4.8	0.08
3	QL2 + fox + fox*QL2	10	8,679.6	7.3	0.02
4	QL2	8	8,680.4	8.0	0.02
EB + VH + QL2 + rabbit + rabbit*EB + rabbit*VH + rabbit*QL2	14	8,681.3	8.9	
EB + QL2 + QL2*EB	10	8,681.7	9.4	
5	Intercept	7	8,681.7	9.4	<0.01
QL2 + rabbit + rabbit*QL2	10	8,681.8	9.4	
EB	8	8,682.3	10.0	
QL1	8	8,682.8	10.4	
EB + fox + fox*EB	10	8,683.0	10.7	
EB + VH + EB*VH	10	8,683.1	10.7	
VH	8	8,683.5	11.1	
Fox	8	8,683.7	11.3	
Rabbit	8	8,683.7	11.4	
QL1 + fox + fox*QL1	10	8,684.1	11.7	
EB + QL1 + QL1*EB	10	8,685.7	13.3	
VH + QL1 + QL1*VH	10	8,686.0	13.7	
EB + rabbit + rabbit*EB	10	8,686.2	13.9	
QL1 + rabbit + rabbit*QL1	10	8,686.4	14.0	
VH + fox + fox*VH	10	8,687.4	15.0	
VH + rabbit + rabbit*VH	10	8,687.4	15.1	
EB + VH + QL1 + fox + fox*EB + fox*VH + fox*QL1	14	8,689.4	17.0	
EB + VH + QL1 + rabbit + rabbit*EB + rabbit*VH + rabbit*QL1	14	8,692.9	20.5	

Notes

Model parameters: QL1 = 1st PCA component of forage quality: N and P; QL2 = 2nd PCA component of forage quality: NDF (+) and Ca(−); EB = edible biomass (g/m^2^); VH = vegetation height (cm); fox, red fox activity; rabbit = rabbit activity. All models contained the control variable area size. Models are based on 979 observations of 11 hare in 20 vegetation types over 71 days.

AICc: Akaike information criterion corrected for small sample size; ∆AICc: delta AICc with regard to best fitting model; *w*_*i*_: Akaike weight or relative weight of each model.

a Only parsimonious models were weighted, that is, more complex models with lower AICc (shaded) were left out.

**Table A3:** Results of the generalized linear mixed model on the effect of predator and competitor activity and its interaction with forage quality and edible biomass on the proportion of time spent foraging of European hares in a vegetation type

Rank	Model type	*df*	AICc	ΔAIC	*w*_*i*_ a
1	QL2 + fox + fox*QL2	12	36,771.8	0.0	0.64
2	QL2 + rabbit + rabbit*QL2	12	36,773.5	1.7	0.27
3	EB + QL2 + QL2*EB	12	36,776.8	5.0	0.05
4	QL2	10	36,778.2	6.4	0.03
	EB + VH + QL2 + fox + fox*EB + fox*VH + fox*QL2	16	36,778.8	7.0	
	EB + VH + QL2 + rabbit + rabbit*EB + rabbit*VH + rabbit*QL2	16	36,779.1	7.3	
	VH + QL2 + QL2*VH	12	36,781.4	9.6	
5	VH + fox + fox*VH	12	36,781.8	10.0	<0.01
	EB + VH + QL1 + fox + fox*EB + fox*VH + fox*QL1	16	36,782.0	10.2	
6	VH + rabbit + rabbit*VH	12	36,783.8	12.0	<0.01
7	EB + VH + EB*VH	12	36,785.9	14.1	<0.01
8	QL1 + fox + fox*QL1	12	36,786.8	15.0	<0.01
	EB + VH + QL1 + rabbit + rabbit*EB + rabbit*VH + rabbit*QL1	16	36,787.2	15.4	
9	Fox	10	36,787.6	15.8	<0.01
10	VH	10	36,787.7	15.9	<0.01
	VH + QL1 + QL1*VH	12	36,790.4	18.6	
	Fox + rabbit + fox*rabbit	12	36,790.4	18.6	
	EB + fox + fox*EB	12	36,791.3	19.5	
11	QL1	10	36,793.9	22.1	<0.01
12	Intercept	9	36,794.0	22.2	<0.01
	Rabbit	10	36,794.7	22.8	
	EB	10	36,795.8	24.0	
	QL1 + rabbit + rabbit*QL1	12	36,796.6	24.8	
	EB + QL1 + QL1*EB	12	36,797.9	26.1	
	EB + rabbit + rabbit*EB	12	36,798.1	26.3	

Notes

Model parameters: QL1 = 1st PCA component of forage quality: N and P; QL2 = 2nd PCA component of forage quality: NDF (+) and Ca(−); EB = edible biomass (g/m^2^); fox = red fox activity; rabbit = rabbit activity. All models contained the control variables area type and sex. Models are based on 2,843 observations of 11 hare in 19 vegetation types in 2 areas over 79 days.

AICc: Akaike information criterion corrected for small sample size; ∆AICc: delta AICc with regard to best fitting model; *w*_*i*_: Akaike weight or relative weight of each model.

a Only parsimonious models were weighted, that is, more complex models with lower AICc (shaded) were left out.
